# Incidence of COVID-19 Hospitalisation in Patients with Systemic Lupus Erythematosus: A Nationwide Cohort Study from Denmark

**DOI:** 10.3390/jcm10173842

**Published:** 2021-08-27

**Authors:** René Cordtz, Salome Kristensen, Louise Plank Holm Dalgaard, Rasmus Westermann, Kirsten Duch, Jesper Lindhardsen, Christian Torp-Pedersen, Lene Dreyer

**Affiliations:** 1 Department of Rheumatology, Aalborg University Hospital, 9000 Aalborg, Denmark; sakr@rn.dk (S.K.); louise.dalgaard@rn.dk (L.P.H.D.); raw@rn.dk (R.W.); k.duch@rn.dk (K.D.); l.dreyer@rn.dk (L.D.); 2 Department of Rheumatology, Center for Rheumatology and Spine Diseases, Gentofte Hospital, 2900 Hellerup, Denmark; 3 Department of Clinical Medicine, Aalborg University, 9000 Aalborg, Denmark; 4 Unit of Clinical Biostatistics, Aalborg University Hospital, 9000 Aalborg, Denmark; 5 Lupus and Vasculitis Clinic, Center for Rheumatology and Spine Diseases, Rigshospitalet, 2100 Copenhagen, Denmark; jesper.lindhardsen@regionh.dk; 6 Department of Cardiology, Nordsjællands Hospital, 3400 Hillerod, Denmark; christian.torp-pedersen.01@regionh.dk; 7 Department of Public Health, University of Copenhagen, 1014 Copenhagen, Denmark

**Keywords:** systemic lupus erythematosus, COVID-19, hydroxychloroquine, glucocorticoids

## Abstract

Background: Patients with systemic lupus erythematosus (SLE) have an increased risk of infections due to impaired immune functions, disease activity, and treatment. This study investigated the impact of having SLE on the incidence of hospitalisation with COVID-19 infection. Methods: This was a nationwide cohort study from Denmark between 1 March 2020 to 2 February 2021, based on the linkage of several nationwide registers. The adjusted incidence of COVID-19 hospitalisation was estimated for patients with SLE compared with the general population in Cox-regression models. Among SLE patients, the hazard ratio (HR) for hospitalisation was analysed as nested case-control study. Results: Sixteen of the 2533 SLE patients were hospitalised with COVID-19 infection. The age-sex adjusted rate per 1000 person years was 6.16 (95% CI 3.76–10.08) in SLE patients, and the corresponding hazard ratio was 2.54 (95% CI 1.55–4.16) compared with the matched general population group after adjustment for comorbidities. Among SLE patients, hydroxychloroquine treatment was associated with a HR for hospitalisation of 0.61 (95% CI 0.19–1.88), and 1.06 (95% CI 0.3–3.72) for glucocorticoid treatment. Conclusion: Patients with SLE were at increased risk of hospitalisation with COVID-19.

## 1. Introduction

Patients with systemic lupus erythematosus (SLE) are considered at higher risk of infections compared with the general population owing to SLE-related innate immune perturbations and the use of immunosuppressive drugs [1]. This raised the question about whether patients with SLE might be at increased risk for contracting SARS-CoV-2 and a more severe clinical course once infected. In a cross-sectional survey study of 165 SLE patients from the Lombardy and Emilia-Romagna regions of Italy, 2% had confirmed coronavirus disease 2019 (COVID-19) [2]. The corresponding proportions in the background population were lower (0.76 and 0.47% in Lombardy and Emilia-Romagna, respectively), but as the authors pointed out, a potential bias could be a higher test frequency among SLE patients although no information was available to disprove or confirm such a tendency. In a prior study describing the incidence and severity of COVID-19 hospitalisation in patients with inflammatory rheumatic diseases in Denmark during the first wave of the epidemic, we found a statistically non-significantly 40% increased risk of hospital admission in patients with connective tissue disease including SLE patients [3]. Only a few studies have investigated the risk of COVID hospitalisation specifically in patients with SLE. In a survey study by Ramirez et al., 417 patients with SLE responded, and the frequency of COVID-19 hospitalisation was 0.24%, compared to 0.43% in the general population of Lombardy [4]. However, the information provided by the responders could not be verified and the reported proportions were not age- and sex-standardised.

In the first period of the COVID-19 pandemic, treatment with hydroxychloroquine (HCQ) was suggested as an inhibitor of SARS-CoV-2 in vitro, but recent studies involving SLE patients have demonstrated that the doses used in treatment of SLE are not protective against severe COVID-19 infection [5,6,7,8,9,10]. On the other hand glucocorticoids might increase the risk of hospitalisation and a subsequent severe outcome [11,12]. Furthermore, studies suggest that pausing the SLE treatment during COVID-19 infection leads to a flare up in the SLE [13,14,15].

Using the nationwide registers in Denmark, this study aimed to investigate the impact of having SLE on the incidence of hospitalisation with COVID-19 infection compared with the general population, and secondarily aimed at investigating the potential association between treatment with HCQ or glucocorticoids and the risk of being hospitalised with COVID-19 among SLE patients.

## 2. Materials and Methods

### 2.1. Study Design

This was a population-based observational cohort study investigating the incidence of COVID-19 hospitalisation in patients with SLE from 1 March 2020 to 2 February 2021, based on the linkage of several Danish nationwide registers.

### 2.2. Data Sources, Study Population and Exposures

The Danish Civil Registration System contains information on all residents of Denmark including the unique civil registration number, which allows for linkage between registers. The Civil Registration System was used to identify the primary cohort consisting of all individuals aged 18 years or older alive on 1 March 2020 of the entire Danish population [16], and to obtain information on age and sex, used for the matching procedures, and vital status during follow-up.

SLE patients were identified from the Danish National Patient Register (DNPR) using the International Classification of Diseases 10th Edition (ICD-10) code M32, except M32.0, according to the algorithm suggested by Hermansen et al., 2016 [17,18]. This case definition requires that a first registration of SLE in the DNPR should be followed by (a) 1 year of out-patient follow up or (b) consecutive inpatient admissions coded with an SLE diagnosis with over 3-month intervals during the first year of follow up. Patients who fulfilled either (a), (b), or both prior to 1 March 2020 constituted the SLE group. Each SLE patient was matched with up to 1000 individuals of the same age and sex from the general population corresponding to the onset of the COVID-19 epidemic in Denmark, 1 March 2020, while requiring that the matched controls had no history of inflammatory rheumatic diseases.

For descriptive purposes, information on redeemed prescriptions of HCQ, azathioprine, methotrexate, glucocorticoid, warfarin, clopidogrel, and acetylsalicylic acid was obtained from the Danish National Database of Reimbursed Prescriptions (DNDRP) using Anatomical Therapeutic Chemical Classification System (ATC)-codes [19] within 1 year prior to 1 March 2020. In the nested case control study, information on redeemed prescriptions of HCQ and glucocorticoid in the 6 months leading up to date of hospitalisation for cases, and matching dates for corresponding controls, was obtained using the DNDRP. Information on treatment with rituximab, belimumab, cyclophosphamide, and mycophenolate mofetil in a hospital setting was obtained from DNPR within 1 year prior to 1 March 2020.

### 2.3. Outcome Information

COVID-19 hospitalisation was obtained through DNPR using ICD-10 codes created by the Danish Ministry of Health specifically for the pandemic in accordance with the definition established by the World Health Organization (ICD-10 codes B34.2A, B97.2 and B97.2A). These codes have recently been validated in a Danish setting [20]. Hospitalisation was defined as a registration with the abovementioned ICD-10 codes and with a further requirement that the hospital-stay lasted at least 24 h.

Secondarily, the number of patients experiencing a severe outcome of COVID-19 hospitalisation was obtained for the SLE and the general population comparator group. A severe outcome was defined as the composite of either acute respiratory distress syndrome, admission to an intensive care unit, and/or death.

### 2.4. Other Covariates

Chronic lung disease, cardiovascular disease (ischemic heart disease, heart failure, hypertension, and stroke), diabetes mellitus type I and II (DM), hospital registered diagnosis of obesity, and cancer were chosen as comorbidities of interest. These diagnoses were identified using ICD-10 codes in the DNPR and/or redeemed prescriptions registered in the Danish Prescription Register of relevant drugs for each comorbidity (see Supplementary Table S1) [21].

In the nested case-control analysis, lupus nephritis was used as a matching parameter to account for SLE disease severity among cases and controls. Lupus nephritis was defined according to the register-based definition suggested by Hermansen et al., which had a positive predictive value of 90% for lupus nephritis. Thus, we identified patients registered with a nephritis diagnosis (ICD-10 codes N00-06, N08.2, N08.5, N16.2, N16.4, N16.8, N18, N19, N26, M32.1B) in the DNPR between the date of SLE diagnosis and date of case/control matching.

### 2.5. Statistical Analysis

The incidence of hospitalisation in SLE patients compared with the general population was found by following the cohort from 1 March 2020 to 2 February 2021, the date of a COVID-19 hospitalisation or date of death, whichever occurred first. For each group, the age- and sex standardised incidence rate of hospitalisation per 1000 person years was estimated. The incidence of hospitalisation with COVID-19 in SLE patients was compared with the matched general population group in a Cox-regression model with age as underlying time scale, and stratified by sex to estimate a hazard ratio (HR) with 95% confidence interval (95%CI). An additional Cox model was adjusted for the following comorbidities: chronic lung disease, DM, cardiovascular disease, obesity, and cancer.

Furthermore, a Cox model with covariates age (restricted cubic spline with four degrees of freedom), sex, and group (SLE vs. general population) was used to estimate the predicted probability of being hospitalised with COVID-19 stratified according to age (40-, 60-, and 80-year-olds), sex, and SLE or non-SLE status. The predicted probability was plotted with cumulative absolute risk of COVID-19 hospitalisation in % on the *y*-axis and months since start of the pandemic in Denmark (1 March 2020) on the *x*-axis.

Lastly, in a nested case-control design of the SLE patients, each SLE patient admitted with COVID-19 (cases) was matched with up to five SLE patients who were not hospitalised (controls) on age (3-year intervals), sex, time at risk during the pandemic, and history of nephritis corresponding to the date of COVID-19 hospitalisation for cases and controls. Baseline characteristics were presented with count and percentage for discrete variables unless fewer than three patients were observed. Continuous variables were presented with median and interquartile range (IQR). HR was calculated using conditional logistic regression to account for the dependence of matching and presented with 95% CI. Both separate crude analyses for HCQ and glucocorticoid, and subsequently an adjusted model including both HCQ and glucocorticoid were performed.

## 3. Results

From 1 March 2020, 2533 individuals with SLE were followed up; 88.5% were women with a median age of 55.4 years (Table 1). SLE patients had higher prevalence of all comorbidities compared with the matched general population group. 

Of the 2533 patients with SLE, 46.2% were treated with HCQ and 28.9% received glucocorticoids with the corresponding proportions in the general population group being 0.1 and 3.6%, respectively.

Sixteen of the 2533 SLE patients were hospitalised with COVID-19 infection during follow-up, while the corresponding number of patients in the general population group was 5069 (0.63% vs. 0.20%), Table 2. The age- and sex-standardised incidence rate was threefold higher in SLE patients than in the general population. The age- and sex-adjusted HR was 3.23 (95% CI 1.98 to 5.28). The estimate was slightly attenuated following adjustment for comorbidities: 2.54 (95% CI 1.55 to 4.16).

**Figure 1:**
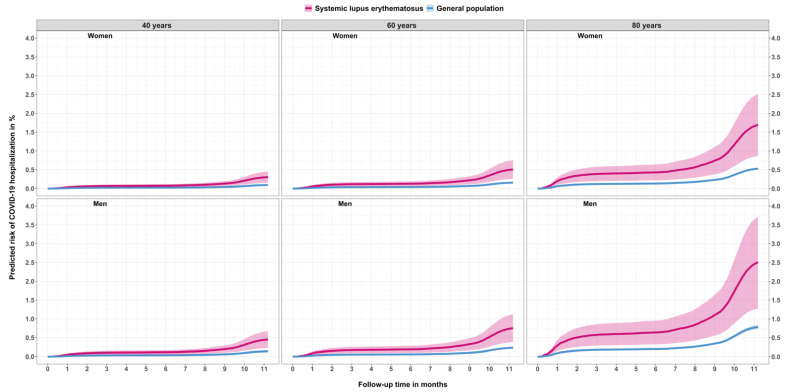
Predicted risk of COVID-19 hospitalisation in % follow-up in months for patients with systemic lupus erythematosus and the general population stratified by sex and age.

The predicted cumulative incidence increased with age and was higher among men than women. Also, the incidence was higher for SLE patients compared with the general population across all age and sex specific strata (Figure 1).

The proportion of patients experiencing a severe outcome during their COVID-19 hospitalisation was similar between the SLE and the general population groups, but due to GPDR regulations, the absolute number of events was too low to present here.

In the nested case-control analyses, the adjusted HR for COVID-19 hospitalisation among SLE patients treated with HCQ was 0.61 (95% CI 0.19 to 1.88) compared with non-HCQ treated patients, whereas the corresponding HR for glucocorticoid treated compared with non-glucocorticoid treated SLE patients was 1.06 (95% CI 0.30 to 3.72) (see Table 3).

## 4. Discussion

This nationwide study showed an increased risk of hospitalisation with COVID-19 for the 2533 SLE patients compared with the age- and sex-matched group from the background population. Among the SLE patients, there was no association between HCQ nor glucocorticoid treatment and the risk of being hospitalised with COVID-19.

Several case reports and small case series on SLE and COVID-19 have been published, but to date, only few cohort- or registry-based studies focusing on the incidence of hospitalisation of patients with SLE infected with COVID-19 exist.

To ensure sufficient data on COVID-19 in rheumatic patients, the Global Rheumatology Alliance established a COVID-19 register with support from the ACR and EULAR, allowing clinicians to register information on patients with rheumatic disease and COVID-19 [22]. In a report of the first 600 patients, there were 85 SLE patients registered. In unadjusted chi-squared analysis, differences in hospitalisation status by disease revealed that a higher percentage of the cohort who were hospitalised had SLE (17%) versus those who were not hospitalised (11%). However, given the mechanism of collection of the case information and the mix of rheumatic diseases in the cohort, it is impossible to conclude if the SLE patients in that study were more likely to be admitted with COVID-19 than non-SLE individuals.

In a French study based on hospitalisation data of all inpatients during the first 6 months of the pandemic 1411 patients with SLE were hospitalised with COVID-19 [23]. Among these 17% needed treatment in intensive care unit and 9.5% died. Furthermore, severe infection in patients with SLE was associated with age, male gender, and comorbidities such as hypertension and chronic kidney disease. However, the population was not compared with age- and sex-matched individuals from the general population. In this study only few patients suffered from severe COVID-19 infection and cannot be concluded on.

In the beginning of the COVID-19 pandemic it was suggested that treatment with HCQ could have a prophylactic effect on infection with SARS-CoV2 based on in vitro studies [24]. However, large in vivo studies have since shown that routine treatment with HCQ in patients with rheumatic disease does not protect against infection nor hospitalisation with SARS-CoV2 [25,26] *per se*. Similarly, we found no association between routine treatment with HCQ and a lower likelihood of being admitted with COVID-19.

In a previous study, of 17 HCQ-treated patients with SLE, 71% were taking glucocorticoids, most of them below 10 mg prednisone equivalent, and 41% received other immunosuppressants [12]. Almost half of these patients were admitted with COVID-19 to intensive care and 2 of the 17 patients died. These findings along with others [6] have raised the question whether long-term glucocorticoid treatment might have played a role.

In the present study, having redeemed a prescription for glucocorticoid in the 6 months leading up to the index date for cases and controls was not associated with increased risk of hospital admission in SLE patients. We recognise that the absolute number of patients treated with HCQ and glucocorticoids in the present study is too low for any clear association to be identified, but overall, there was no indication of either protective or harmful effects of HCQ and glucocorticoid treatment in the SLE group.

Strengths: The main strengths of the present study are the complete follow-up of a nationwide cohort of patients with SLE using a validated identification algorithm for SLE in combination with the use of registers with high degree of completeness. Additionally, the overall high positive predictive value of 99% (95% CI 99–100) for COVID-19 hospital diagnosis, which was consistently high among all subgroups of sex, age groups, and calendar period, is another important strength [20]. Lastly, the ability to create an age- and sex-matched comparator group is a strength of the present study, as one of the most consistent problems in the existing literature on SLE and COVID-19 has been the lack of non-rheumatic comparator groups or insufficient age- and sex-standardised rates and proportions when comparing with the general population.

Limitations: It is possible that patients with SLE are admitted with a lower threshold than people from the general population due to their immunosuppressed state, although we believe this to be less likely due to the outcome definition that excluded patients who stayed <24 h in-hospital. Rather, we may have underestimated the true risk of hospitalisation related to COVID-19 infection in patients with SLE, as these patients potentially exhibit more behavioural social distancing precautions compared with the general population and therefore are less likely to contract COVID-19. Indeed, a Danish study reported that patients with inflammatory rheumatic diseases self-isolated more than others of the same age, and it is likely to be the same for patients with SLE [27]. Using ICD-10 codes for case definition could potentially be a limitation; however, as mentioned, the ICD-10 codes for SLE and COVID-19 in the DNPR have been validated with high PPVs [17,20]. Also, information regarding serologic parameters and disease activity could have been of interest to study predictors of hospitalisation and severe outcome, yet such information could not be collected.

Another limitation is that our dataset was rather small in terms of outcomes (hospitalisations) among SLE patients, especially when it came to specific treatment types for SLE. Furthermore, no information on kidney and lung function was available, and nor could we collect specific information regarding the prothrombotic state that patients with SLE experience due to antiphospholipid syndromes and other potential SLE-intrinsic prothrombotic risk factors [28]. It cannot be ruled out that the increased incidence of hospitalisation in SLE patients could to some extent be explained by either of these factors.

## 5. Conclusions

In this unselected nationwide cohort of Danish SLE patients, there was an approximately threefold increased incidence of hospitalisation with COVID-19 compared with age- and sex-matched controls from the general population after adjustment for several confounders. There was no obvious impact on the risk of hospitalisation associated with glucocorticoid nor HCQ treatment in this cohort, but the number of hospitalisations was too low to draw any definite conclusion, and we encourage further investigations into whether SLE in itself, as well as specific SLE treatment modalities, pose any modulation of risk for both hospitalisation and/or developing poor subsequent outcomes of COVID-19 infection.

## Figures and Tables

**Table 1:** Demographics, comorbidities, and medication in SLE and the general population at the start of follow-up.

Group	Systemic Lupus Erythematosus	General Population
*n*	2533	2,532,914
Age in years, median (interquartile range)	55.4 (44.1 to 66.5)	55.5 (44.1 to 66.6)
Women, *n* (%)	2242 (88.5%)	2,241,914 (88.5%)
Disease duration in years, median (interquartile range)	12.6 (6.1 to 21.7)	-
Lupus nephritis, *n* (%)	205 (8.1%)	-
Cardiovascular disease, *n* (%)	1070 (42.2%)	447,760 (17.7%)
Lung disease, *n* (%)	595 (23.5%)	330,635 (13.1%)
Diabetes mellitus, *n* (%)	202 (8.0%)	165,528 (6.5%)
Cancer, *n* (%)	221 (8.7%)	201,063 (7.9%)
Diagnosed with obesity, *n* (%)	300 (11.8%)	251,883 (9.9%)
Treated with, *n* (%)		
Hydroxychloroquine	1170 (46.2%)	1830 (0.1%)
Azathioprine	202 (8.0%)	3418 (0.1%)
Methotrexate	118 (4.7%)	7282 (0.3%)
Glucocorticoids	685 (27.0%) ^1^	67,738 (2.7%)
Cyclophosphamide	8 (0.3%)	439 (0%)
Mycophenolate mofetil	54 (2.1%)	336 (0%)
Rituximab	42 (1.7%)	1494 (0.1%)
Belimumab	30 (1.2%)	0 (0%)
Warfarin	306 (12.1%)	21,613 (0.9%)
Clopidogrel	142 (5.6%)	68,210 (2.7%)
Acetylsalicylic acid	440 (17.4%)	136,380 (5.4%)

^1^ Based on redeemed glucocorticoid-prescriptions 12 months prior to index, the average daily dosage redeemed was estimated to: 0–5 mg 45.5%, 5–10 mg 41.3% and ≥10 mg 13.2%. SLE: systemic lupus erythematosus.

**Table 2:** Numbers, incidence rates and hazard ratios for hospitalisation with COVID-19 infection among SLE patients and the general population.

Analysis	Systemic Lupus Erythematosus	General Population
*N* hospitalised with COVID-19	16	5069
Person years of observation	2616.7	2,634,850.9
Age- and sex-adjusted rates per 1000 person years (95% CI)	6.16 (3.76 to 10.08)	1.91 (1.86 to 1.96)
HR (95% CI) for hospitalisation with COVID-19 adjusted for sex with age as underlying time scale	3.20 (1.96 to 5.24)	1 (Reference)
HR (95% CI) for hospitalisation with COVID-19 adjusted for sex and comorbidities with age as underlying time scale	2.62 (1.55 to 4.16)	1 (Reference)

*N*: Numbers, 95% CI: 95% Confidence Interval; HR, hazard ratio.

**Table 3:** Numbers, incidence rates and hazard ratios for hospitalisation with COVID-19 infection among hospitalised patients with SLE matched with controls from the SLE population.

Group	SLE Cases Hospitalised with COVID-19	Matched Controls from SLE Population not Hospitalised with COVID-19
*N*	16	79
Age in years, median (interquartile range)	69.1 (55.5–78.8)	67.3 (52.6 to 78.9)
Women, *n* (%)	11 (68.8%)	55 (69.9%)
Disease duration in years, median (interquartile range)	12.6 (9.9–22.3)	14.9 (5.6–23.9)
Lupus nephritis, *n* (%)	≤3	15 (19%)
Cardiovascular disease, *n* (%)	7 (43.8%)	22 (27.8%)
Lung disease, *n* (%)	5 (31.2%)	15 (19%)
Diabetes Mellitus, *n* (%)	4 (25%)	13 (16.5%)
Cancer, *n*	≤3	≤3
Diagnosed with obesity, *n*	≤3	≤3
Hydroxychloroquine, *n* (%)	5 (31.2%)	34 (43%)
Glucocorticoids, *n* (%)	4 (25%)	19 (24.1%)
Crude HR (95% CI) for COVID-19 hospitalisation in hydroxychloroquine treated compared with non- hydroxychloroquine treated	0.61 (0.19 to 1.88)	1 (Reference)
Crude HR (95% CI) for COVID-19 hospitalisation in glucocorticoid treated compared with non- glucocorticoid treated	1.06 (0.30 to 3.72)	1 (Reference)
Adjusted * HR (95% CI) for COVID-19 hospitalisation in hydroxychloroquine treated compared with non- hydroxychloroquine treated	0.60 (0.19 to 1.87)	1 (Reference)
Adjusted * HR (95% CI) for COVID-19 hospitalisation in glucocorticoid treated compared with non- glucocorticoid treated	1.12 (0.32 to 3.96)	1 (Reference)

*N*: numbers, OR: odds ratio, CI: confidence interval. * Matched on age (3-year intervals), sex, time at risk, and history of lupus nephritis/yes/no), and model with both glucocorticoid (yes/no) and hydroxychloroquine treatment (yes/no).

## Data Availability

According to Danish legislation, none of the original data can be shared.

## Supplementary Materials

The following are available online at https://www.mdpi.com/article/10.3390/jcm10173842/s1, Table S1: Definition of comorbidities.
