# Supplementary material for: Incidence of COVID-19 Hospitalisation in Patients with Systemic Lupus Erythematosus: A Nationwide Cohort Study from Denmark
Source: J Clin Med. 2021 Aug 27;10(17):3842. doi: 10.3390/jcm10173842 (PMC8432052; doi:10.3390/jcm10173842)
Supplement: Supplementary file 1 [file jcm-10-03842-s001.zip › jcm-1341861-supplementary.pdf]

Table S1. Definition of comorbidities.

Case definitions are based on<br>1) ICD-10 entries in the Danish National Patient Registry with 1 or more ICD-10 codes prior to baseline<br>and/or<br>2) ATC codes in the Danish National Prescription Register with prescriptions filled within 1 years from baseline.	
Chronic lung disease	ICD-10 codes: J4x or J84x or<br>ATC codes: R03Ax or RO3Bx
Diabetes mellitus	ICD-10 codes: E10-14 or<br>ATC codes: A19x
Cardiovascular disease	ICD-10 codes. I1x, I2x, I50x, I6x
Obesity	ICD-10 codes: E66x
Cancer	ICD-10 codes: Cx excl. C44 (non- melanoma skin cancer)

ICD-10: international classification of diseases 10th edition; ATC: anatomical therapeutic chemical classification system.
